# Primary endometrioid stromal sarcomas of the ovary: a clinicopathological study of 14 cases with a review of the literature

**DOI:** 10.18632/oncotarget.18805

**Published:** 2017-06-28

**Authors:** Weimin Xie, Xiaoning Bi, Dongyan Cao, Jiaxin Yang, Keng Shen, Yan You

**Affiliations:** ^1^ Department of Obstetrics and Gynecology, Peking Union Medical College Hospital, Chinese Academy of Medical Sciences and Peking Union Medical College, Beijing 100730, China; ^2^ Department of Pathology, Peking Union Medical College Hospital, Chinese Academy of Medical Sciences and Peking Union Medical College, Beijing 100730, China

**Keywords:** ovarian endometrioid stromal sarcoma, low-grade, high-grade, recurrence, prognosis

## Abstract

**Purpose:**

Primary endometrioid stromal sarcomas (ESS) of the ovary are rare mesenchymal tumors with scarce data on their behavior and optimal treatment. We aimed to describe the clinicopathologic features and outcome among patients with primary ovarian ESS.

**Results:**

The age of the patients ranged from 34 to 61 years (mean: 49.1 years, median: 51.5 years). The most common symptoms were abdominal distention or pain or both. Nine (64.3%) and five patients (35.7%) had low-grade and high-grade disease, respectively. The median duration of follow-up was 65 months (range, 8–311 months). All 9 patients with low-grade ESS were alive, of these, 3 (33.3%) of them developed recurrence after surgery. Only 1 patient (20%) with high-grade ESS was alive with no evidence of disease in a short-term follow-up visit; the remaining 4 (80%) developed recurrence after surgery, and 2 (40%) died of progressive disease.

**Methods:**

Medical records of 14 patients with primary ovarian ESS in our institution were collected and analyzed.

**Conclusions:**

The behavior of primary ovarian ESS is similar to that of their uterine counterparts. Low-grade ESS is an indolent tumor with a propensity for late recurrences. The prognosis of high-grade ESS is poor.

## INTRODUCTION

Endometrial stromal sarcomas are rare mesenchymal tumors accounting for approximately 0.2% of female genital tract malignancies [1, 2]. These mesenchymal neoplasms occur most commonly in the uterus and occasionally originate from extrauterine sites, such as the ovary, bowel wall, peritoneum, pelvis, and vagina [2, 3]. Extrauterine endometrial stromal sarcomas are conventionally referred to as “endometrioid stromal sarcomas (ESS)”, which are mesenchymal tumors identical to uterine endometrial stromal sarcomas. Endometrial stromal sarcomas of the uterus are well-known uterine neoplasms and recent advances have been made in molecular genetics. In contrast, experience with primary ESS of the ovary is limited because of their rarity. Less than 100 cases of ovarian ESS have been reported to date [1–19], and the inclusion criteria of both primary and metastatic tumors, in some case series, has made it difficult to confirm features specific to primary ovarian cases [2–4]. The newly released 2014 WHO classification divides these tumors into two different subtypes based on pathologic features: low-grade ESS and high-grade ESS [20]. Herein, we present the clinicopathological features of 14 primary ovarian ESS from a single institution, including 9 low-grade ESS and 5 high-grade ESS.

## RESULTS

### Clinical features

The clinicopathologic features of the patients are summarized in Table 1. The age of the 14 patients ranged from 34 to 61 years (mean: 49.1 years, median: 51.5 years). The most common symptoms were abdominal distention or pain or both, which was reported in 10 of the 14 patients (71.4%). One patient (7.1%) presented with dysmenorrhea. Three patients (21.4%) were asymptomatic, and the tumors were discovered incidentally by ultrasonography on routine examination. Three patients had previously undergone hysterectomy for benign gynecologic conditions. Serum CA125 level was evaluated for 13 patients before the operation. Six patients showed elevated CA125 level, and 4 of them had endometriosis.

**Table 1:** Clinicopathologic features of 14 cases with primary ovarian endometrioid stromal sarcomas

Case	Age,y	Laterality	Size	Grade	Stage	Treatment	RFS(mo)	Location ofrecurrence	Status(mo)	Status uterus
1	40	Right	8	LG	III	TH+BSO, L, A, tumor debulking, RT, HT	–	–	NED(55)	Serosa involved
2	54	Left	5	LG	III	BSO, tumor debulking, RT, CT	252	Retroperitoneum	AWD(311)	Myoma
3	40	Left	8	LG	III	TH+BSO, L, A, tumor debulking, CT, HT	–	–	NED(76)	Negative
4	43	Right	18	LG	I	TH+BSO	–	–	NED(140)	Negative
5	34	Bilateral	4, 8	LG	III	TH+BSO, L, tumor debulking, CT	–	–	NED(48)	Negative
6	61	Right	14	LG	I	BSO, A, CT	–	–	NED(54)	Myoma
7	60	Left	5	LG	I	TH+BSO, A, HT	–	–	NED(121)	Negative
8	43	Right	Unk	LG	I	TH+RSO	30	Left ovary, Rectum,	NED(64)	Negative
9	50	Bilateral	Unk	LG	III	BSO, tumor debulking, CT	8	Rectum	NED(66)	Negative
10	59	Left	Unk	HG	I	TH+BSO, L, A, CT	22	Pelvis, rectum	AWD(72)	Negative
11	53	Right	15	HG	III	BSO, tumor debulking, CT	–	–	NED(8)	Myoma
12	61	Bilateral	Unk	HG	III	TH+BSO, L, A, tumor debulking, CT	24	Pelvis, colon	DOD(112)	Negative
13	34	Right	Unk	HG	I	TH+RSO, A, CT	12	Pelvis, sigmoid colon, rectum	DOD(18)	Negative
14	55	Right	8	HG	III	TH+BSO, L, A, tumor debulking, CT	8	Pelvis, colon, liver	AWD(17)	Serosa involved

LG, low-grade; HG, high-grade; TH, total hysterectomy; BSO, bilateral salpingo-oophorectomy; RSO, right salpingo-oophorectomy; L, lymph node dissection; A, appendicectomy; RT, radiotherapy; CT, chemotherapy; HT, hormonal therapy; RFS, recurrence-free survival; NED, no evidence of disease; AWD, Alive with disease; DOD, dead of disease; Unk, unknown.

### Treatment

Eight patients underwent total hysterectomy with bilateral salpingo-oophorectomy. Three patients who had previously undergone hysterectomy for uterine myomas were treated by bilateral salpingo-oophorectomy. The remaining three patients who were referred to our hospital with a recurrence were initially treated by bilateral salpingo-oophorectomy (1), or hysterectomy with unilateral salpingo-oophorectomy (2) at outside hospitals. In 12 patients, additional procedures including omentectomy, appendicectomy, lymph node dissection, debulking of extraovarian tumor, and peritoneal biopsies were performed. The tumor was unilateral in 11 cases and bilateral in 3 cases. Of the 14 patients, 6 had stage I disease, and 8 had stage III disease according to the 2009 International Federation of Gynecology and Obstetrics staging system.

### Pathologic features

Gross findings of the primary surgery were available for 9 cases. The greatest diameter of the tumors ranged from 4 to 18 cm (average, 9.5 cm). Seven tumors were solid, and 2 were solid cystic. The sectioned surfaces of the solid areas were tan or yellow-white and had a moderate or soft consistency.

**Figure 1:**
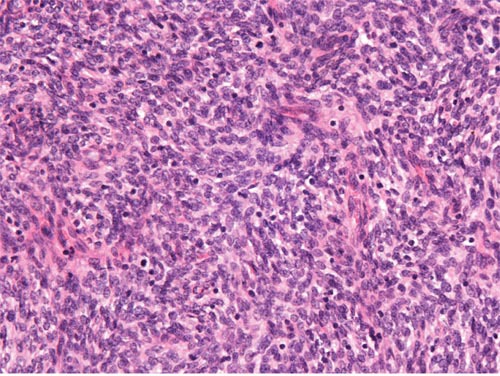
Low-grade ESS The tumor is composed of generally uniform cells with scant cytoplasm and round to oval nuclei (original magnification × 400, hematoxylin-eosin stain).

**Figure 2:**
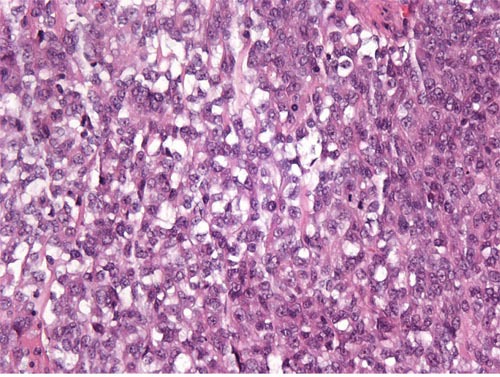
High-grade ESS The tumor is composed of atypical round cells. (original magnification × 400, hematoxylin-eosin stain).

On microscopic examination, 9 cases showed features of low-grade ESS. The tumors were typically composed of sheets of small, closely pack cells resembling the stromal cells of the proliferative endometrium, with scant cytoplasm and round to oval nuclei (Figure 1). Sex cord-like differentiation was observed focally in 2 cases. Five cases showed features of high-grade ESS. The tumors were characterized by a monomorphic proliferation of round cells, which were larger than those of low-grade ESS with increased cytoplasm and high-grade cytologic atypia (Figure 2). Three of the five cases of high-grade ESS had a component of low-grade ESS.

**Figure 3:**
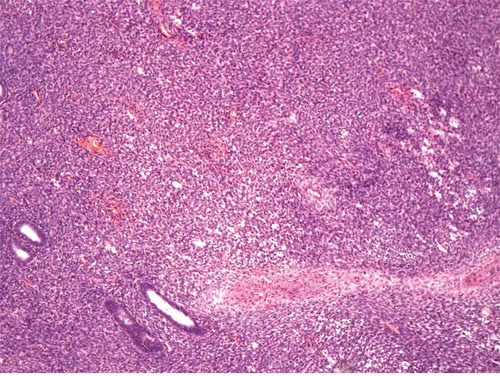
A few endometrioid glands are observed in the tumor (original magnification × 100, hematoxylin-eosin stain)

The tumor was associated with endometriosis in 6 cases (Figure 3). Sections of the uterus were evaluated in 14 cases. There was no evidence of endometrial stromal sarcomas in 12 patients. In two, the serous membrane was involved directly by the tumor, but the endometrium was normal. According to the operative records and pathologic findings, omentum metastasis was found in 2 (22.2%, 2/9) cases, rectum metastasis in 3 (100%, 3/3) cases, and appendix metastasis in 1 (12.5%, 1/8) case; no pelvic lymph node metastasis was found in 6 cases.

The immunohistochemical features of low-grade ESS were different from those of high-grade ESS. In low-grade ESS, estrogen receptor (ER) was positive in 5/7 tumors evaluated, progesterone receptor (PR) in 6/7, and CD10 in 7/8, while α-inhibin was negative in 2/2 tumors evaluated, desmin in 3/3, and caldesmon in 3/3. In the 5 cases of high-grade ESS, ER was focal positive in 2 cases, PR and CD10 were focal positive in 3 cases. Cyclin D1 was diffusely positive in 2 cases tested. The following markers were negative in those tumors tested: α-inhibin (1), calretinin (1), desmin (2).

### Adjuvant therapy

Of the 9 cases with low-grade ESS, 7 were treated with adjuvant therapy after the initial surgery. Three cases received chemotherapy and one received hormonal therapy alone, one received chemotherapy in combination with hormonal therapy. The remaining two cases received radiotherapy combined with chemotherapy or hormonal therapy, respectively. The chemotherapy regimens included PEI (cisplatin + epirubicin + ifosfamide), VAC (vincristine + actinomycin + cyclophosphamide), and TC (paclitaxel + carboplatin). The average number of chemotherapy courses for low-grade ESS was 4.2 (range 3-6). All the 5 cases with high-grade ESS were treated with adjuvant chemotherapy after the initial surgery, including PEI, PAC (cisplatin + epirubicin + cyclophosphamide), and gemcitabine plus docetaxel. The average number of chemotherapy courses for high-grade ESS was 5 (range 5-8).

### Follow-up

The median duration of follow-up was 65 months (range, 8–311 months). Of the 9 cases with low-grade ESS, 3 (33.3%) developed recurrence at 8, 30, and 252 months after surgery, respectively. All the recurrences were intra-abdominal and treated with tumor debulking and adjuvant therapy. All 9 patients are living: 8 were alive and free of disease and one was alive with disease. However, only 1 case (20%) with high-grade ESS was alive with no evidence of disease in a short-term (8 month) follow-up visit; the remaining four (80%) developed recurrence at 8, 12, 22, and 24 months after surgery, respectively. All patients who had recurrences were treated with tumor debulking and adjuvant chemotherapy, with or without hormonal therapy or radiotherapy. At the date of the investigation, 2 (40%) died of progressive disease and 2 (40%) was alive with disease.

## DISCUSSION

Endometrial stromal sarcomas are rare tumors of endometrial stromal origin that usually involve the endometrium and myometrium. Primary extrauterine ESS are rare mesenchymal tumors showing evidence of endometrial stromal differentiation without indication of uterine origin. The ovary is one of the most common extrauterine locations according to our experience and previous studies. Primary ovarian ESS are divided into low-grade and high-grade subtypes based on microscopic features and molecular findings according the newly released 2014 WHO classification system. Based on previous reports, high-grade ESS has the same clinical presentation but different prognosis compared to low-grade ESS. The incidence of high-grade ESS of the ovary is much lower than that of low-grade subtype. To the best of our knowledge, this study represents the largest series of primary ovarian high-grade ESS reported to date.

Consistent with previous reports [4, 5], the present study showed that most of primary ovarian ESS occurred in perimenopausal women. Patients commonly presented with nonspecific symptoms such as abdominal distention or pain or both, but some were asymptomatic. By contrast, patients with uterine low-grade and high-grade endometrial stromal sarcoma usually present with abnormal vaginal bleeding or pelvic pain [21]. Serum CA125 level was only increased in 6 out of 13 tumors evaluated, which was less sensitive than epithelial ovarian cancer. In addition, 4 of 6 tumors with elevated CA125 level had endometriosis, making the value of CA125 confusing. Primary ovarian ESS are typically unilateral, as seen in 78.6% (11/14) of our series. On the contrary, most of ovarian metastases from uterine endometrial stromal sarcomas are bilateral [22]. According to previous reports, approximately half of primary ovarian ESS are associated with endometriosis, and it is widely hypothesized that the primary extrauterine ESS including ovarian ESS may originate from the stroma of ectopic endometrium [19, 23, 24]. As the majority of endometrial stromal sarcomas are intrauterine, the status of the uterus should be fully evaluated to exclude the possibility of ovarian metastasis. Unless the uterus is known to be negative on pathological examination, it may be impossible to differentiate primary ovarian ESS from ovarian metastasis because neither bilaterality nor ovarian endometriosis can be given much weight in the differential [4, 5].

On microscopic examination, primary ovarian ESS, like their more common uterine counterparts, are divided to low-grade ESS and high-grade ESS based on tumor differentiation and resemblance to proliferative endometrial stroma. Low-grade ESS is typically characterized by sheets of cells resembling the stromal cells of proliferative endometrium and small blood vessels resembling the spiral arterioles of late secretory endometrium [20]. Several histologic variants including smooth muscle differentiation, sex cord-like differentiation, fibrous or myxoid change, glandular differentiation may occur and pose diagnostic challenges [2, 4, 5, 20, 25]. In the present study, the typical microscopic pattern was seen in all 9 cases and sex cord-like differentiation was identified in 2 cases. The important differential diagnoses include sex cord-stromal tumors, particularly adult granulosa cell tumor, fibroma/fibrosarcoma, low-grade mullerian adenosarcoma, endometriosis, smooth muscle tumors, and metastatic gastrointestinal stromal tumor (GIST) [2, 5, 20, 25]. In our series, two tumors were initially misdiagnosed as sex cord-stromal tumors on frozen-section examination. High-grade ESS demonstrates only modest endometrial stromal differentiation with high mitotic activity [20]. The important differential diagnoses include low-grade ESS, epithelioid leiomyosarcoma, mullerian adenosarcoma, sex cord-stromal tumors, and metastatic GIST [4, 21]. Previous reports, in addition to our own experiences, have indicated that a component of low-grade ESS can be seen in some cases of high-grade ESS and may cause diagnostic difficulties [26, 27].

In difficult cases, immunohistochemistry is often employed to make the distinction. Low-grade ESS characteristically shows diffuse positivity for CD10, ER, and PR, while high-grade ESS typically shows absent or only focal and weak staining for CD10, ER and PR, diffuse positivity for cyclin D1 [21]. Although CD10 is helpful in distinguishing fibroma and fibrosarcoma from ESS, it is nonspecific for ESS and may be identified in a variety of tumors, including smooth muscle tumors and sex cord stromal tumors [21, 28]. An immunohistochemical panel that includes CD10 and at least two smooth muscle markers such as desmin, caldesmon, and HDAC8 is helpful in the differential diagnosis of smooth muscle tumors from ESS [29]. Distinguishing low-grade ESS from sex cord-stromal tumors in the ovary can represent a diagnostic challenge. The typical area of low-grade ESS lacks expression of α-inhibin and calretinin and a panel consisting of CD10, calretinin, and α-inhibin may prove helpful in this distinction [2, 25]. High-grade ESS shows strong and diffuse positivity for cyclin D1, which is particularly useful in the differential diagnosis [26, 30]. In our series, cyclin D1 was diffusely positive in 2 cases tested. Recently, some typical genetic alterations of uterine low-grade endometrial stromal sarcoma, including JAZF-SUZ12 gene fusion and PHF1 gene rearrangement, have been identified in ovarian low-grade ESS [20]. However, the YWHAE-FAM22 gene fusion resulting from translocation t(10;17)(q22;p13) that is seen in the corresponding uterine tumor has not been reported in ovarian high-grade ESS [20].

Based on our experience and a review of the available literature, surgical resection is the mainstay of treatment for primary ovarian ESS. Primary surgery for early disease includes hysterectomy and bilateral salpingo-oophorectomy; tumor debulking is reserved for advanced-stage disease [1, 11, 20]. Lymph node metastasis can occur in uterine endometrial stromal sarcomas at a rate of 2% to 45%; however, lymphadenectomy does not seem to improve survival [26, 31, 32]. The rate of lymph node metastasis in primary ovarian ESS is unknown owing to the rarity of these tumors. In our study, none of the patients with primary ovarian ESS had evidence of lymph node involvement. However, the small series do not permit us to draw any conclusions, and the role of lymphadenectomy in the treatment of primary ovarian ESS remains elusive.

Similar to the treatment for the comparable uterine tumors, adjuvant therapies including radiotherapy, chemotherapy, and hormonal therapy have been used for the management of primary ovarian ESS [1, 4, 6, 11, 20]. Low-grade ESS is considered a hormone-dependent malignancy, which means hormonal therapy with high-dose oral progestins, gonadotropin-releasing hormone analogs, and aromatase inhibitors may be effective. Progestins are considered to be beneficial and are widely used for residual or recurrent low-grade ESS. However, prospective data evaluating the role of hormone therapy are lacking. Adjuvant radiotherapy appears to be useful for both low-grade and high-grade ESS in reducing the risk of local recurrence, however, no studies to date have been able to show a survival advantage associated with the use of adjuvant radiotherapy [4, 31]. The role of chemotherapy in the management of both low-grade and high-grade ESS of the ovary is unclear. The optimal choice of chemotherapy regimen has not yet been determined. The varied use of chemotherapy regimens for the small series in our study limited further analysis on their roles in adjuvant therapy.

The prognosis of primary ovarian ESS is similar to that of their uterine counterparts. Low-grade ESS is a slow-growing malignant neoplasm with an indolent clinical course, which is characterized by late recurrences and favorable prognosis [20, 26]. High-grade ESS is an aggressive tumor that is associated with a poor prognosis [4, 20]. In the present study, all 9 cases of low-grade ESS were alive and 33.3% of them developed recurrence after surgery. It is worth noting that one case showed a late recurrence at 252 months, thus, long-term follow-up is required. The recurrence and death rates of high-grade ESS were 80% and 40%, respectively. Compared with low-grade ESS, patients with high-grade ESS showed earlier and more frequent recurrences and were less likely to respond to the salvage systemic therapy.

In conclusion, primary ovarian ESS are extremely rare malignant neoplasms. Accurate diagnoses should be made cautiously and metastatic uterine sarcomas of the same cell type should be excluded. Currently, there is no well-established consensus regarding optimal treatment strategies for these patients. Surgical resection is the mainstay of treatment, and the value of adjuvant therapy remains elusive. The behavior of primary ovarian ESS is similar to that of their uterine counterparts with tumor differentiation being of major prognostic significance. Low-grade ESS is an indolent tumor with a propensity for late recurrences, requiring long-term follow-up for these patients. High-grade ESS is an aggressive tumor with early recurrences and poor outcomes. The rarity of primary ovarian ESS mandates the need for prospective, multicenter, randomized trials to better inform and establish the optimal treatment for these patients.

## MATERIALS AND METHODS

We searched the surgical pathology files of Peking Union Medical College Hospital to identify patients diagnosed with primary ovarian ESS between March 1990 and December 2015. Clinical information including age at the time of diagnosis, clinical features, surgical findings, pathological findings, recurrence patterns, treatment modality, and follow-up data were collected from the medical records and telephone calls. Operation notes and pathology reports were reviewed to determine the sites of the tumors and the extent of surgical staging. Tumor stage was assessed using the 2009 International Federation of Gynecology and Obstetrics staging system. All eligible patients’ pathological slides were reviewed and confirmed by two independent pathologists.

We found 15 cases of primary ovarian ESS from the preliminary search and excluded 1 case of synchronous ESS and undifferentiated sarcoma of the ovary. Nine cases of low-grade ESS and 5 cases of high-grade ESS were identified according to the following criteria. Confirmations of the original site and the microscopic diagnosis were necessary for the inclusion criteria. Patients who had the largest mass(es) located in the ovary without involvement of the uterus were considered primary ovarian ESS, while patients who had concurrent or prior uterine endometrial stromal sarcomas were excluded. The microscopic diagnosis of primary ovarian ESS was established according to the 2014 WHO classification criteria [20]: low-grade ESS was characterized by small, closely pack cells resembling the stromal cells of the proliferative endometrium; high-grade ESS had to be only modest endometrial stromal differentiation without marked nuclear pleomorphism as seen in undifferentiated sarcoma. Cases of undifferentiated sarcoma and other sarcomas, such as carcinosarcoma and leiomyosarcoma of the ovary, were excluded. Immunohistochemistry was applied to assist in the distinction of ESS for challenging cases with unclear morphologic distinction.

The study protocol was reviewed and approved by the ethics committee of Peking Union Medical College Hospital, and informed consent was obtained from all patients for data collection and publication.
